# An Overview of Heart Rate Variability Metrics and Norms

**DOI:** 10.3389/fpubh.2017.00258

**Published:** 2017-09-28

**Authors:** Fred Shaffer, J. P. Ginsberg

**Affiliations:** ^1^Center for Applied Psychophysiology, Truman State University, Kirksville, MO, United States; ^2^William Jennings Bryan Dorn VA Medical Center (VHA), Columbia, SC, United States

**Keywords:** biofeedback, complexity, heart rate variability, non-linear measurements, normative values, optimal performance

## Abstract

Healthy biological systems exhibit complex patterns of variability that can be described by mathematical chaos. Heart rate variability (HRV) consists of changes in the time intervals between consecutive heartbeats called interbeat intervals (IBIs). A healthy heart is not a metronome. The oscillations of a healthy heart are complex and constantly changing, which allow the cardiovascular system to rapidly adjust to sudden physical and psychological challenges to homeostasis. This article briefly reviews current perspectives on the mechanisms that generate 24 h, short-term (~5 min), and ultra-short-term (<5 min) HRV, the importance of HRV, and its implications for health and performance. The authors provide an overview of widely-used HRV time-domain, frequency-domain, and non-linear metrics. Time-domain indices quantify the amount of HRV observed during monitoring periods that may range from ~2 min to 24 h. Frequency-domain values calculate the absolute or relative amount of signal energy within component bands. Non-linear measurements quantify the unpredictability and complexity of a series of IBIs. The authors survey published normative values for clinical, healthy, and optimal performance populations. They stress the importance of measurement context, including recording period length, subject age, and sex, on baseline HRV values. They caution that 24 h, short-term, and ultra-short-term normative values are not interchangeable. They encourage professionals to supplement published norms with findings from their own specialized populations. Finally, the authors provide an overview of HRV assessment strategies for clinical and optimal performance interventions.

## Heart Rate Variability

Heart rate is the number of heartbeats per minute. Heart rate variability (HRV) is the fluctuation in the time intervals between adjacent heartbeats (1). HRV indexes neurocardiac function and is generated by heart-brain interactions and dynamic non-linear autonomic nervous system (ANS) processes. HRV is an emergent property of interdependent regulatory systems which operate on different time scales to help us adapt to environmental and psychological challenges. HRV reflects regulation of autonomic balance, blood pressure (BP), gas exchange, gut, heart, and vascular tone, which refers to the diameter of the blood vessels that regulate BP, and possibly facial muscles (2).

A healthy heart is not a metronome. The oscillations of a healthy heart are complex and non-linear. A healthy heart’s beat-to-beat fluctuations are best described by mathematical chaos (3). The variability of non-linear systems provides the flexibility to rapidly cope with an uncertain and changing environment (4). While healthy biological systems exhibit spatial and temporal complexity, disease can involve either a loss or increase of complexity (5).

Higher HRV is not always better since pathological conditions can produce HRV. When cardiac conduction abnormalities elevate HRV measurements, this is strongly linked to increased risk of mortality (particularly among the elderly). Close examination of electrocardiogram (ECG) morphology can reveal whether elevated HRV values are due to problems like atrial fibrillation (6).

An optimal level of HRV is associated with health and self-regulatory capacity, and adaptability or resilience. Higher levels of resting vagally-mediated HRV are linked to performance of executive functions like attention and emotional processing by the prefrontal cortex (1). Afferent information processing by the intrinsic cardiac nervous system can modulate frontocortical activity and impact higher-level functions (7).

## A Brief Overview of HRV Metrics

We can describe 24 h, short-term (ST, ~5 min) or brief, and ultra-short-term (UST, <5 min) HRV using time-domain, frequency-domain, and non-linear measurements. Since longer recording epochs better represent processes with slower fluctuations (e.g., circadian rhythms) and the cardiovascular system’s response to a wider range of environment stimuli and workloads, short-term and ultra-short-term values are not interchangeable with 24 h values.

*Time-domain* indices of HRV quantify the amount of variability in measurements of the interbeat interval (IBI), which is the time period between successive heartbeats (see Table 1). These values may be expressed in original units or as the natural logarithm (Ln) of original units to achieve a more normal distribution (8).

**Table 1:** HRV time-domain measures.

Parameter	Unit	Description
SDNN	ms	Standard deviation of NN intervals
SDRR	ms	Standard deviation of RR intervals
SDANN	ms	Standard deviation of the average NN intervals for each 5 min segment of a 24 h HRV recording
SDNN index (SDNNI)	ms	Mean of the standard deviations of all the NN intervals for each 5 min segment of a 24 h HRV recording
pNN50	%	Percentage of successive RR intervals that differ by more than 50 ms
HR Max − HR Min	bpm	Average difference between the highest and lowest heart rates during each respiratory cycle
RMSSD	ms	Root mean square of successive RR interval differences
HRV triangular index		Integral of the density of the RR interval histogram divided by its height
TINN	ms	Baseline width of the RR interval histogram

*Interbeat interval, time interval between successive heartbeats; NN intervals, interbeat intervals from which artifacts have been removed; RR intervals, interbeat intervals between all successive heartbeats*.

*Frequency-domain* measurements estimate the distribution of absolute or relative power into four frequency bands. The Task Force of the European Society of Cardiology and the North American Society of Pacing and Electrophysiology (1996) divided heart rate (HR) oscillations into ultra-low-frequency (ULF), very-low-frequency (VLF), low-frequency (LF), and high-frequency (HF) bands (see Table 2).

**Table 2:** HRV frequency-domain measures.

Parameter	Unit	Description
ULF power	ms^2^	Absolute power of the ultra-low-frequency band (≤0.003 Hz)
VLF power	ms^2^	Absolute power of the very-low-frequency band (0.0033–0.04 Hz)
LF peak	Hz	Peak frequency of the low-frequency band (0.04–0.15 Hz)
LF power	ms^2^	Absolute power of the low-frequency band (0.04–0.15 Hz)
LF power	nu	Relative power of the low-frequency band (0.04–0.15 Hz) in normal units
LF power	%	Relative power of the low-frequency band (0.04–0.15 Hz)
HF peak	Hz	Peak frequency of the high-frequency band (0.15–0.4 Hz)
HF power	ms^2^	Absolute power of the high-frequency band (0.15–0.4 Hz)
HF power	nu	Relative power of the high-frequency band (0.15–0.4 Hz) in normal units
HF power	%	Relative power of the high-frequency band (0.15–0.4 Hz)
LF/HF	%	Ratio of LF-to-HF power

*Power* is the signal energy found within a frequency band. Frequency-domain measurements can be expressed in absolute or relative power. *Absolute power* is calculated as ms squared divided by cycles per second (ms^2^/Hz). *Relative power* is estimated as the percentage of total HRV power or in normal units (nu), which divides the absolute power for a specific frequency band by the summed absolute power of the LF and HF bands. This allows us to directly compare the frequency-domain measurements of two clients despite wide variation in specific band power and total power among healthy, age-matched individuals (9).

The *ULF band* (≤0.003 Hz) indexes fluctuations in IBIs with a period from 5 min to 24 h and is measured using 24 h recordings (10). The *VLF band* (0.0033–0.04 Hz) is comprised of rhythms with periods between 25 and 300 s. The *LF band* (0.04–0.15 Hz) is comprised of rhythms with periods between 7 and 25 s and is affected by breathing from ~3 to 9 bpm. Within a 5 min sample, there are 12–45 complete periods of oscillation (9). The *HF or respiratory band* (0.15–0.40 Hz) is influenced by breathing from 9 to 24 bpm (11). The *ratio of LF to HF power (LF/HF ratio)* may estimate the ratio between sympathetic nervous system (SNS) and parasympathetic nervous system (PNS) activity under controlled conditions. *Total power* is the sum of the energy in the ULF, VLF, LF, and HF bands for 24 h and the VLF, LF, and HF bands for short-term recordings (12).

Finally, *non-linear measurements* (see Table 3) allow us to quantify the unpredictability of a time series (13).

**Table 3:** HRV non-linear measures.

Parameter	Unit	Description
S	ms	Area of the ellipse which represents total HRV
SD1	ms	Poincaré plot standard deviation perpendicular the line of identity
SD2	ms	Poincaré plot standard deviation along the line of identity
SD1/SD2	%	Ratio of SD1-to-SD2
ApEn		Approximate entropy, which measures the regularity and complexity of a time series
SampEn		Sample entropy, which measures the regularity and complexity of a time series
DFA α1		Detrended fluctuation analysis, which describes short-term fluctuations
DFA α2		Detrended fluctuation analysis, which describes long-term fluctuations
D_2_		Correlation dimension, which estimates the minimum number of variables required to construct a model of system dynamics

## Sources of HRV

This section explores the sources of short-term and 24 h HRV measurements. The authors will not separately discuss ultra-short-term HRV measurements since they are controversial proxies for short-term HRV values and there is an absence of research concerning their physiological origin.

## Short-Term HRV

Two distinct but overlapping processes generate short-term HRV measurements. The first source is a complex and dynamic relationship between the sympathetic and parasympathetic branches. The second source includes the regulatory mechanisms that control HR *via* respiratory sinus arrhythmia (RSA), the baroreceptor reflex (negative-feedback control of BP), and rhythmic changes in vascular tone (2). *RSA* refers to the respiration-driven speeding and slowing of the heart *via* the vagus nerve (14).

### Dynamic Autonomic Relationship

In a healthy human heart, there is a dynamic relationship between the PNS and SNS. PNS control predominates at rest, resulting in an average HR of 75 bpm. The PNS can slow the heart to 20 or 30 bpm, or briefly stop it (15). This illustrates the response called *accentuated antagonism* (16).

Parasympathetic nerves exert their effects more rapidly (<1 s) than sympathetic nerves (>5 s) (17). Since these divisions can produce contradictory actions, like speeding and slowing the heart, their effect on an organ depends on their current balance of activity. While the SNS can suppress PNS activity, it can also increase PNS reactivity (18). *Parasympathetic rebound* may occur following high levels of stress, resulting in increased nighttime gastric activity (19) and asthma symptoms (20).

The relationship between the PNS and SNS branches is complex (both linear and non-linear) and should not be described as a “zero sum” system illustrated by a teeter-totter. Increased PNS activity may be associated with a decrease, increase, or no change in SNS activity. For example, immediately following aerobic exercise, HR recovery involves PNS reactivation while SNS activity remains elevated (21, 22).

Likewise, teaching clients to breathe slowly when they experience high levels of SNS activity can engage both branches and increase RSA. This is analogous to a Formula 1^®^ driver speeding through a turn while gently applying the left foot to the brake, a maneuver called “left-foot braking.” The complex relationship between SNS and PNS nerve activity means that the ratio between LF and HF power will not always index autonomic balance (21).

### Regulatory Mechanisms

The autonomic, cardiovascular, central nervous, endocrine, and respiratory systems, and baroreceptors and chemoreceptors influence HRV over a short time period and contribute to the very-low to high frequencies of the HRV spectrum (12). Baroreceptors, which are BP sensors located in the aortic arch and internal carotid arteries, contribute to short-term HRV (23). When you inhale, HR increases. BP rises about 4–5 s later. Baroreceptors detect this rise and fire more rapidly. When you exhale, HR decreases. BP falls 4–5 s later (24, 25). The baroreflex makes this acceleration and deceleration of the heart, called RSA, possible (14).

The baroreflex links HR, BP, and vascular tone. Oscillation in one cardiovascular function causes identical oscillations in the others (26). Baroreceptor firing due to BP changes activates mechanisms that change HR and vascular tone. Rising BP triggers decreases in HR and vascular tone, while falling BP causes increases in both.

## Twenty-Four-Hour HRV

Circadian rhythms, core body temperature, metabolism, the sleep cycle, and the renin–angiotensin system contribute to 24 h HRV recordings, which represent the “gold standard” for clinical HRV assessment (12). These recordings achieve greater predictive power than short-term measurements (10, 27–29). Although we calculate 24 h and short-term HRV measurements using the same mathematical formulas, they cannot substitute for each other and their physiological meaning can profoundly differ (9).

## Time-Domain Measurements

Heart rate variability time-domain indices quantify the amount of HRV observed during monitoring periods that may range from <1 min to >24 h. These metrics include the SDNN, SDRR, SDANN, SDNN Index, RMSSD, NN50, pNN50, HR Max − HR Min, the HRV triangular index (HTI), and the Triangular Interpolation of the NN Interval Histogram (TINN, see Table 1). Where appropriate, the authors reported accepted minimum short-term and proposed ultra-short-term measurement periods.

### SDNN

The standard deviation of the IBI of normal sinus beats (SDNN) is measured in ms. "Normal" means that abnormal beats, like ectopic beats (heartbeats that originate outside the right atrium’s sinoatrial node), have been removed. While the conventional short-term recording standard is 5 min (11), researchers have proposed ultra-short-term recording periods from 60 s (30) to 240 s (31). The related standard deviation of successive RR interval differences (SDSD) only represents short-term variability (9).

Both SNS and PNS activity contribute to SDNN and it is highly correlated with ULF, VLF and LF band power, and total power (32). This relationship depends on the measurement conditions. When these bands have greater power than the HF band, they contribute more to SDNN.

In short-term resting recordings, the primary source of the variation is parasympathetically-mediated RSA, especially with slow, paced breathing (PB) protocols (12). In 24 h recordings, LF band power makes a significant contribution to SDNN (9). The SDNN is more accurate when calculated over 24 h than during the shorter periods monitored during biofeedback sessions. Longer recording periods provide data about cardiac reactions to a greater range of environmental stimulation. In addition to cardiorespiratory regulation, extended measurement periods can index the heart’s response to changing workloads, anticipatory central nervous activity involving classical conditioning, and circadian processes, including sleep-wake cycles. Twenty-four-hour recordings reveal the SNS contribution to HRV (33).

The SDNN is the "gold standard" for medical stratification of cardiac risk when recorded over a 24 h period (11). SDNN values predict both morbidity and mortality. Based on 24 h monitoring, patients with SDNN values below 50 ms are classified as unhealthy, 50–100 ms have compromised health, and above 100 ms are healthy (34). Heart attack survivors, whose 24 h measurements placed them in a higher category, had a greater probability of living during a 31-month mean follow-up period. For example, patients with SDNN values over 100 ms had a 5.3 times lower risk of mortality at follow-up than those with values under 50 ms (34). Does this mean that training patients to increase SDNN to a higher category could reduce their risk of mortality?

### SDRR

The standard deviation of the IBIs for all sinus beats (SDRR), including abnormal or false beats, is measured in ms. As with the SDNN, the SDRR measures how these intervals vary over time and is more accurate when calculated over 24 h because this longer period better represents slower processes and the cardiovascular system’s response to more diverse environmental stimuli and workloads. Abnormal beats may reflect cardiac dysfunction or noise that masquerades as HRV.

### SDANN

The standard deviation of the average normal-to-normal (NN) intervals for each of the 5 min segments during a 24 h recording (SDANN) is measured and reported in ms like the SDNN. This refers to IBIs calculated after artifacting the data. SDANN is not a surrogate for SDNN since it is calculated using 5 min segments instead of an entire 24 h time series (9) and it does not provide additional useful information (12).

### SDNN Index (SDNNI)

The SDNNI is the mean of the standard deviations of all the NN intervals for each 5 min segment of a 24-h HRV recording. Therefore, this measurement only estimates variability due to the factors affecting HRV within a 5-min period. It is calculated by first dividing the 24 h record into 288 5 min segments and then calculating the standard deviation of all NN intervals contained within each segment. The SDNNI is the average of these 288 values. The SDNNI primarily reflects autonomic influence on HRV. The SDNNI correlates with VLF power over a 24-h period (12).

### NN50

The number of adjacent NN intervals that differ from each other by more than 50 ms (NN50) requires a 2 min epoch.

### pNN50

The percentage of adjacent NN intervals that differ from each other by more than 50 ms (pNN50) also requires a 2-min epoch. Researchers have proposed ultra-short-term periods of 60 s (31). The pNN50 is closely correlated with PNS activity (32). It is correlated with the RMSSD and HF power. However, the RMSSD typically provides a better assessment of RSA (especially in older subjects) and most researchers prefer it to the pNN50 (35). This may be a more reliable index than short-term SDNN measurements for the brief samples used in biofeedback.

### RMSSD

The root mean square of successive differences between normal heartbeats (RMSSD) is obtained by first calculating each successive time difference between heartbeats in ms. Then, each of the values is squared and the result is averaged before the square root of the total is obtained. While the conventional minimum recording is 5 min, researchers have proposed ultra-short-term periods of 10 s (30), 30 s (31), and 60 s (36).

The RMSSD reflects the beat-to-beat variance in HR and is the primary time-domain measure used to estimate the vagally mediated changes reflected in HRV (12). The RMSSD is identical to the non-linear metric SD1, which reflects short-term HRV (37). Twenty-four-hour RMSSD measurements are strongly correlated with pNN50 and HF power (27). Minimum HR is more strongly correlated with LnSDANN than LnRMSSD (Ln means the natural logarithm). Maximum HR is weakly and inconsistently correlated with these time-domain measures (38).

While the RMSSD is correlated with HF power (10), the influence of respiration rate on this index is uncertain (39, 40). The RMSSD is less affected by respiration than is RSA across several tasks (41). The RMSSD is more influenced by the PNS than SDNN. Lower RMSSD values are correlated with higher scores on a risk inventory of sudden unexplained death in epilepsy (42).

NN50, pNN50, and RMSSD are calculated using the differences between successive NN intervals. Since their computation depends on NN interval differences, they primarily index HF HR oscillations, are largely unaffected by trends in an extended time series, and are strongly correlated (9).

### HR Max − HR Min

The average difference between the highest and lowest HRs during each respiratory cycle (HR Max − HR Min) is especially sensitive to the effects of respiration rate, independent of vagus nerve traffic. At least a 2-min sample is required to calculate HR Max − HR Min. Instead of directly indexing vagal tone, it reflects RSA. Since longer exhalations allow greater acetylcholine metabolism, slower respiration rates can produce higher RSA amplitudes that are not mediated by changes in vagal firing.

### HRV Triangular Index

The HTI is a geometric measure based on 24 h recordings which calculates the integral of the density of the RR interval histogram divided by its height (11). A 5-min epoch is conventionally used to represent this metric (43). HTI and RMSSD can jointly distinguish between normal heart rhythms and arrhythmias. When HTI ≤ 20.42 and RMSSD ≤ 0.068, the heart rhythm is normal. When HTI > 20.42, the pattern is arrhythmic (43).

### Triangular Interpolation of the NN Interval Histogram

The TINN is the baseline width of a histogram displaying NN intervals (11). Like SDNN and RMSSD, contamination by only two artifacts within a 5-min segment can significantly distort its value (8).

## Frequency-Domain Measurements

Analogous to the electroencephalogram, we can use Fast Fourier Transformation (FFT) or autoregressive (AR) modeling to separate HRV into its component ULF, VLF, LF, and HF rhythms that operate within different frequency ranges. This is analogous to a prism that refracts light into its component wavelengths (11).

## ULF Band

The ultra-low-frequency (ULF) band (≤0.003 Hz) requires a recording period of at least 24 h (12) and is highly correlated with the SDANN time-domain index (44). While there is no consensus regarding the mechanisms that generate ULF power, very slow-acting biological processes are implicated. Circadian rhythms may be the primary driver of this rhythm (12). Core body temperature, metabolism, and the renin–angiotensin system operate over a long time period and may also contribute to these frequencies (11, 45). There is disagreement about the contribution by the PNS and SNS to this band. Different psychiatric disorders show distinct circadian patterns in 24 h HRs, particularly during sleep (46, 47).

## VLF Band

The VLF band (0.0033–0.04 Hz) requires a recording period of at least 5 min, but may be best monitored over 24 h. Within a 5-min sample, there are about 0–12 complete periods of oscillation (9). While all low values on all 24 h clinical HRV measurements predict greater risk of adverse outcomes, VLF power is more strongly associated with all-cause mortality than LF or HF power (48–51). The VLF rhythm may be fundamental to health (12).

Low VLF power has been shown to be associated with arrhythmic death (44) and PTSD (52). Low power in this band has been associated with high inflammation in several studies (53, 54). Finally, low VLF power has been correlated with low levels of testosterone, while other biochemical markers, such as those mediated by the hypothalamic–pituitary–adrenal axis (e.g., cortisol), have not (55).

Very-low-frequency power is strongly correlated with the SDNNI time-domain measure, which averages 5 min standard deviations for all NN intervals over a 24-h period. There is uncertainty regarding the physiological mechanisms responsible for activity within this band (10). The heart’s intrinsic nervous system appears to contribute to the VLF rhythm and the SNS influences the amplitude and frequency of its oscillations (12).

Very-low-frequency power may also be generated by physical activity (56), thermoregulatory, renin–angiotensin, and endothelial influences on the heart (57, 58). PNS activity may contribute to VLF power since parasympathetic blockade almost completely abolishes it (59). In contrast, sympathetic blockade does not affect VLF power and VLF activity is seen in tetraplegics, whose SNS innervation of the heart and lungs is disrupted (11, 60).

Based on work by Armour (61) and Kember et al. (62, 63), the VLF rhythm appears to be generated by the stimulation of afferent sensory neurons in the heart. This, in turn, activates various levels of the feedback and feed-forward loops in the heart’s intrinsic cardiac nervous system, as well as between the heart, the extrinsic cardiac ganglia, and spinal column. This experimental evidence suggests that the heart intrinsically generates the VLF rhythm and efferent SNS activity due to physical activity and stress responses modulates its amplitude and frequency.

## LF Band

The LF band (0.04–0.15 Hz) is typically recorded over a minimum 2 min period (12). This region was previously called the baroreceptor range because it mainly reflects baroreceptor activity during resting conditions (1). LF power may be produced by both the PNS and SNS, and BP regulation *via* baroreceptors (11, 57, 64, 65), primarily by the PNS (66), or by baroreflex activity alone (67). The SNS does not appear to produce rhythms much above 0.1 Hz, while the parasympathetic system can be observed to affect heart rhythms down to 0.05 Hz (20 s rhythm). In resting conditions, the LF band reflects baroreflex activity and not cardiac sympathetic innervation (12).

During periods of slow respiration rates, vagal activity can easily generate oscillations in the heart rhythms that cross over into the LF band (68–70). Therefore, respiratory-related efferent vagally mediated influences are particularly present in the LF band when respiration rates are below 8.5 bpm or 7 s periods (70, 71) or when one sighs or takes a deep breath.

## HF Band

The HF or respiratory band (0.15–0.40 Hz) is conventionally recorded over a minimum 1 min period. For infants and children, who breathe faster than adults, the resting range can be adjusted to 0.24–1.04 Hz (72). The HF band reflects parasympathetic activity and is called the respiratory band because it corresponds to the HR variations related to the respiratory cycle. These phasic HR changes are known as RSA and may not be a pure index of cardiac vagal control (73).

Heart rate accelerates during inspiration and slows during expiration. During inhalation, the cardiovascular center inhibits vagal outflow resulting in speeding the HR. Conversely, during exhalation, it restores vagal outflow resulting in slowing the HR *via* the release of acetylcholine (74). Total vagal blockage virtually eliminates HF oscillations and reduces power in the LF range (12).

High-frequency power is highly correlated with the pNN50 and RMSSD time-domain measures (10). HF band power may increase at night and decrease during the day (1). Lower HF power is correlated with stress, panic, anxiety, or worry. The modulation of vagal tone helps maintain the dynamic autonomic regulation important for cardiovascular health. Deficient vagal inhibition is implicated in increased morbidity (75).

### HF Power and RSA do not Represent Vagal Tone

In healthy individuals, RSA can be increased by slow, deep breathing. Respiration rate changes can produce large-scale shifts in RSA magnitude without affecting vagal tone, which is mean HR change across conditions (e.g., rest to exercise) (76). Grossman (76) proposed an experiment. If you slow your breathing to 6 bpm, you should observe increased HR fluctuations compared with 15 bpm. During this time, mean HR should not appreciably change because vagal tone did not change.

While HF power indexes vagal modulation of HR, it does not represent vagal tone. If shifts in HF power mirrored shifts in vagal tone, they should produce corresponding changes in average HR. But, breathing at different rates within the 9–24 bpm range, which changes HF power, does not change mean HR. RSA and vagal tone are dissociated during large-scale changes in SNS activity, chemical blockade of the SA node, and when intense vagal efferent traffic dramatically slows HR during inhalation and exhalation (73). Shifts in respiration rate and volume can markedly change HRV indices (HF power, RSA, pNN50, RMSSD) without actually affecting vagal tone.

### LnHF can Estimate Vagal Tone under Controlled Conditions

The natural logarithm (Ln) is the logarithm to the base e of a numeric value. Under controlled conditions while breathing at normal rates, we can use LnHF power to estimate vagal tone (77).

## LF/HF Ratio

The ratio of LF to HF power (LF/HF ratio) was originally based on 24 h recordings, during which both PNS and SNS activity contribute to LF power, and PNS activity primarily contributes to HF power. The intent was to estimate the ratio between SNS and PNS activity (12).

The assumptions underlying the LF/HF ratio is that LF power may be generated by the SNS while HF power is produced by the PNS. In this model, a low LF/HF ratio reflects parasympathetic dominance. This is seen when we conserve energy and engage in tend-and-befriend behaviors. In contrast, a high LF/HF ratio indicates sympathetic dominance, which occurs when we engage in fight-or-flight behaviors or parasympathetic withdrawal.

Billman (21) challenged the belief that the LF/HF ratio measures “sympatho-vagal balance” (78, 79). First, LF power is not a pure index of SNS drive. Half of the variability in this frequency band is due to the PNS and a smaller proportion is produced by unspecified factors. Second, PNS and SNS interactions are complex, non-linear, and frequently non-reciprocal. Third, confounding by respiration mechanics and resting HR creates uncertainty regarding PNS and SNS contributions to the LF/HF ratio during the measurement period.

Shaffer et al. (12) warned that the LF/HF ratio is controversial because different processes appear to generate 24 h and 5 min values, and these values correlate poorly. Furthermore, the SNS contribution to LF power varies profoundly with testing conditions. For example, when LF is calculated while sitting upright during resting conditions, the primary contributors are PNS activity and baroreflex activity—not SNS activity (63, 80). Therefore, interpretation of 5 min resting baseline LF/HF ratios depends on specific measurement conditions.

## Non-Linear Measurements

From Schrödinger’s (81) perspective, life is aperiodic (e.g., oscillations occur without a fixed period) and operates between randomness and periodicity. Twenty-four-hour ECG monitoring yields a time series of R–R intervals (time period between successive heartbeats). Non-linearity means that a relationship between variables cannot be plotted as a straight line. Non-linear measurements index the unpredictability of a time series, which results from the complexity of the mechanisms that regulate HRV. Non-linear indices correlate with specific frequency- and time-domain measurements when they are generated by the same processes. While stressors and disorders like diabetes can depress some non-linear measurements, elevated values do not always signal health. For example, in postmyocardial infarction (post-MI) patients, increased non-linear HRV is an independent risk factor for mortality (82). This section reviews S, SD1, SD2, SD1/SD2, approximate entropy (ApEn), sample entropy (SampEn), detrended fluctuation analysis (DFA) α1 and DFA α2, and D_2_ non-linear measures (see Table 3).

## Poincaré Plot

A Poincaré plot (return map) is graphed by plotting every R–R interval against the prior interval, creating a scatter plot. Poincaré plot analysis allows researchers to visually search for patterns buried within a time series (a sequence of values from successive measurements). Unlike frequency-domain measurements, Poincaré plot analysis is insensitive to changes in trends in the R–R intervals (83).

## S, SD1, SD2, and SD1/SD2

We can analyze a Poincaré plot by fitting an ellipse (curve which resembles a squashed circle) to the plotted points. After fitting the ellipse, we can derive three non-linear measurements, S, SD1, and SD2. The area of the ellipse which represents total HRV (*S*) correlates with baroreflex sensitivity (BRS), LF and HF power, and RMSSD.

The standard deviation (hence SD) of the distance of each point from the *y* = *x* axis (*SD1*), specifies the ellipse’s width. SD1 measures short-term HRV in ms and correlates with baroreflex sensitivity (BRS), which is the change in IBI duration per unit change in BP, and HF power. The RMSSD is identical to the non-linear metric SD1, which reflects short-term HRV (37). SD1 predicts diastolic BP, HR Max − HR Min, RMSSD, pNN50, SDNN, and power in the LF and HF bands, and total power during 5 min recordings (84, 85).

The standard deviation of each point from the *y* = *x* + average R–R interval (*SD2*) specifies the ellipse’s length. SD2 measures short- and long-term HRV in ms and correlates with LF power and BRS (86–89). The ratio of *SD1/SD2*, which measures the unpredictability of the RR time series, is used to measure autonomic balance when the monitoring period is sufficiently long and there is sympathetic activation. SD1/SD2 is correlated with the LF/HF ratio (83, 90).

## Approximate Entropy

Approximate entropy measures the regularity and complexity of a time series. ApEn was designed for brief time series in which some noise may be present and makes no assumptions regarding underlying system dynamics (9). Applied to HRV data, large ApEn values indicate low predictability of fluctuations in successive RR intervals (91). Small ApEn values mean that the signal is regular and predictable (8).

## Sample Entropy

Sample entropy was designed to provide a less biased and more reliable measure of signal regularity and complexity (92). SampEn values are interpreted and used like ApEn and may be calculated from a much shorter time series of fewer than 200 values (9).

## Detrended Fluctuation Analysis

Detrended fluctuation analysis extracts the correlations between successive RR intervals over different time scales. This analysis results in slope α1, which describes brief fluctuations, and slope α2, which describes long-term fluctuations. The short-term correlations extracted using DFA reflect the baroreceptor reflex, while long-term correlations reflect the regulatory mechanisms that limit fluctuation of the beat cycle. DFA is designed to analyze a time series that spans several hours of data (9).

## Correlation Dimension (CD, D_2_)

The CD (D_2_) estimates the minimum number of variables required to construct a model of system dynamics. The more variables required to predict the time series, the greater its complexity. An attractor is a set of values toward which a variable in a dynamic system converges over time. CD measures a system’s attractor dimension, which can be an integer or fractal (9).

## Context is Crucial When Interpreting HRV Measurements

Awareness of the context of recording and subject variables can aid interpretation of both 24 h and short-term HRV measurements. Important contextual factors include recording period length, detection or recording method, sampling frequency, removal of artifacts, respiration, and whether or not there is PB. Important subject variables are age, sex, HR, and health status. In addition, influences of position, movement, recency of physical activity, tasks, demand characteristics, and relationship variables can all affect measurements subtly or even greatly by changing ANS activation, breathing mechanics, and emotions.

## Contextual Factors

### Period Length

The length of the recording period significantly affects both HRV time-domain and frequency-domain measurements (93). Since longer recordings are associated with increased HRV, it is inappropriate to compare metrics like SDNN when they are calculated from epochs of different length (11, 94). Generally, resting values obtained from short-term monitoring periods correlate poorly with 24 h indices and their physiological meanings may differ (9).

### Detection Method

Electrocardiogram and PPG methods yielded discrepancies of less than 6% for most HRV measures and 29.9% for pNN50 in one study (95).

### Sampling Frequency

While a minimum sampling frequency of 500 Hz may be required to detect the R-spike fiducial point of the ECG when RSA amplitude is low, a sampling rate of 125 Hz (93) or 200 Hz (9) may be sufficient when RSA amplitude is normal. Very low RR interval variability, which characterizes some heart failure patients, requires higher sampling rates for adequate temporal resolution (9). Lower sampling rates may threaten the validity of HRV frequency-domain and non-linear indices (96).

### Removal of Artifacts

Visual inspection of the raw BVP or ECG signal can help detect artifacts (e.g., missed or spurious beats). Artifacts can significantly distort both time- and frequency-domain measurements (97). Artifacts increase power in all frequency bands. Missed beats produce greater increases than extra beats since deviation from a missed beat equals the mean heart period versus half the mean heart period for extra beats. The bias introduced by even a single artifact can easily eclipse the 0.5–1.0 Ln effect sizes typically found in psychophysiological research (98). When artifacts are present, researchers can select an artifact-free epoch or manually edit the affected RR intervals (99). When a clean segment is shorter than the recommended length for calculating power within a frequency band, values should be valid as long as it contains at least six full periods of oscillations. For example, estimation of LF power requires at least 2.5 min of clean data (9).

Researchers can replace technical artifacts like missed beats through interpolation based on QRS intervals that precede and follow the contaminated segment. Data analysis software like Kubios (8) can help visualize the raw signal and preserve the original record length and synchrony with other physiological signals (e.g., respiration). The editing of ectopic beats and arrhythmias can be challenging because the resulting changes in stroke volume and cardiac output can affect 10–30 beats instead of the two RR intervals that bracket the abnormal heartbeat (9).

### Respiration

Greater tidal volumes and lower respiration rates increase RSA (12, 100). Increasing respiration depth raised HR Max − HR Min and did not reduce time-domain, frequency-domain, or non-linear HRV measures (101, 102). Increasing or decreasing respiration rate from a client’s resonance frequency, the breathing rate that best stimulates the cardiovascular system, may lower short-term time-domain measurements and LF band power, while raising or lowering HF power, respectively.

The effect of inhalation-to-exhalation (I/E) ratio on HRV time- and frequency-domain measurements remains unclear. Lin et al. (103) reported that breathing at 5.5 bpm with a 5:5 I/E ratio resulted in higher LF power than with a 4:6 ratio. However, the authors failed to confirm that their subjects actually breathed at the required rates and ratios. Zerr et al. (84, 85) studied different I/E ratios (1:2 and 1:1) at 6 bpm and performed manipulation checks on respiration rate and I/E ratio. They found that HRV time- or frequency-domain values were comparable when healthy undergraduates breathed 6 bpm at 1:2 or 1:1 I/E ratios. A replication study by Meehan et al. (101, 102) also found that HRV time- and frequency-domain values were comparable when healthy undergraduates breathed at 6 bpm at 1:2 or 1:1 I/E ratios.

### Paced Breathing

Values obtained during normal breathing and PB can vary significantly (17).

## Subject Variables

### Age

Heart rate variability time-domain measurements decline with age (17, 104–106). Bonnemeier et al. (104) obtained 24 h recordings from 166 healthy volunteers (85 men and 81 women) ages 20–70. They found the most dramatic HRV parameter decrease between the second and third decades. Almeida-Santos et al. (106) obtained 24 h ECG recordings of 1,743 subjects 40–100 years of age. They found a linear decline in SDNN, SDANN, and SDNN index. However, they discovered a U-shaped pattern for RMSSD and pNN50 with aging, decreasing from 40 to 60 and then increasing after age 70.

### Sex

A meta-analysis of 296,247 healthy participants examined 50 HRV measures (107). Women had higher mean HR (smaller RR intervals) and lower SDNN and SDNN index values, especially in 24 h studies, compared to men. They showed lower total, VLF, and LF power, but greater HF power. While women showed relative vagal dominance, despite higher mean HR, men showed relative SNS dominance, despite their lower HR.

### Heart Rate

Faster HRs reduce the time between successive beats and the opportunity for the IBIs to vary. This lowers HRV. Conversely, slower HRs increase the time between adjacent heartbeats and the chance for IBIs to vary. This raises HRV. This phenomenon is called *cycle length dependence* (1). Resting HRs that exceed 90 bpm are associated with elevated risk of mortality (108).

### Health

Time-domain measurements rise with increased aerobic fitness (109, 110). In general, HRV time-domain measurements decline with decreased health (111, 112). Autonomic cardiac dysregulation is a critical process that underlies the manifestation and perpetuation of symptoms broad spectrum symptoms of poor health. HRV has been shown to be useful in predicting morbidities from common mental (e.g., stress, depression, anxiety, PTSD) and physical disorders (e.g., inflammation, chronic pain, diabetes, concussion, asthma, insomnia, fatigue), all of which increase sympathetic output and create a self-perpetuating cycle that produces autonomic imbalance and greater allostatic load (113–121). Thus, ANS dysfunction is a systemic common denominator of poor health and associated with acute and chronic illness and a risk factor for such serious health issues as cancer survivorship, cardiovascular disease and myocardial infarction, stroke, and overall mortality (49, 75, 122–125).

## HRV Norms

### Ultra-Short-Term (UST) Measurement Norms

Ultra-short-term HRV measurements are based on less than 5 min of data (Table 4). Four studies reviewed in this section (31, 126–128) measured HRV during resting baselines while sitting upright or lying supine. One study (30) monitored subjects during resting baseline and Stroop test conditions.

**Table 4:** Ultra-short-term (UST) norms.

Studies	Subjects	HRV monitor	Metrics and minimum epoch required to estimate short-term values
Salahuddin et al. (30)	24 healthy students Age 22–31	ECG	HR and RMSSD-10 s; pNN50, HF (ms^2^ and nu), LF/HF, and LF nu-20 s; LF ms^2^ and VLF ms^2^-50 s; SDNN and the coefficient of variation-60 s; HTI and TINN-90 s to estimate 150 s values

Nussinovitch et al. (126)	70 healthy volunteers Age 42.5 ± 16.1	ECG	10 s and 1 min resting RMSSD values correlated with 5 min RMSSD values, but 10 s and 1 min resting SDNN did not correlate with 5 min SDNN values

Baek et al. (31)	467 healthy volunteers Age 8–69	PPG	HR-10 s; HF ms^2^-20 s; RMSSD-30 s; pNN50-60 s; LF (ms^2^ and nu) and HF nu-90 s; SDNN-240 s; VLF ms^2^-270 s to estimate 5 min values. Minimum values differed by age group

Munoz et al. (129)	3,387 adults (1,727 W and 1,660 M) Mean age 53	Portapres^®^	Near-perfect agreement of 120 s RMSSD and SDNN values with 240–300 s values. UST RMSSD values achieved stronger agreement with 240–300 s values than UST SDNN for all record lengths and agreement metrics (Pearson *r*, Bland-Altman, and Cohen’s *d*)

Shaffer et al. (128)	38 healthy students Age 18–23	ECG	HR-10 s; NN50, and pNN50-60 s; TINN, LF ms^2^, SD1, and SD2-90 s; HTI and DFA ɑ1-120 s; LF nu, HF ms^2^, HF nu, LF/HF, SampEn, DFA ɑ2, and DET-180 s; ShanEn-240 s; VLF ms^2^-270 s to estimate 5 min values. No epoch estimated CD

*Coefficient of variation, ratio of the standard deviation to the mean; CD (also D_2_), correlation dimension, which is the minimum number of variables required to construct a model of system dynamics; DET, determinism of a time series; DFAɑ1, detrended fluctuation analysis, which describes short-term fluctuations; DFA ɑ2, detrended fluctuation analysis, which describes long-term fluctuations; ECG, electrocardiogram; HF ms^2^, absolute power of the high-frequency band; HF nu, relative power of the high-frequency band in normal units; HR, heart rate; HTI, HRV triangular index or integral of the density of the NN interval histogram divided by its height; LF ms^2^, absolute power of the low-frequency band; LF nu, relative power of the low-frequency band in normal units; LF/HF, ratio of LF-to-HF power; NN interval, time between adjacent normal heartbeats; nu, normal units calculated by dividing the absolute power for a specific frequency band by the summed absolute power of the LF and HF bands; pNN50, percentage of successive interbeat intervals that differ by more than 50 ms; RMSSD, root mean square of successive RR interval differences; RR interval, time between all adjacent heartbeats; SampEn, sample entropy, which measures signal regularity and complexity; SD1, Poincaré plot standard deviation perpendicular to the line of identity; SD2, Poincaré plot standard deviation along the line of identity; SDNN, standard deviation of NN intervals; ShanEn, Shannon entropy, which measures uncertainty in a random variable; TINN, triangular interpolation of the RR interval histogram or baseline width of the RR interval histogram; VLF ms^2^, absolute power of the very-low-frequency band*.

The use of ultra-short-term recording to estimate HRV status is important because of its obvious efficiency in both clinical and research settings. However, many of the reviewed ultra-short-term studies (30, 31, 126, 130) suffered from serious methodological limitations. Since only one of the studies (128) specified their minimum criterion for acceptable concurrent validity (e.g., *r* = 0.9), we cannot know the percentage of variability in 5 min values for which their ultra-short-term measurements accounted. Since correlation between measurements doesn’t ensure agreement, the authors recommend that investigators utilize the more rigorous Bland-Altman Limits of Agreement (LoA) method (131, 132) like Munoz et al. (129). This procedure calculates the 95% limits of agreement between two methods of measurement for repeated measures.

Review of this emerging literature suggests that differences in contextual factors such as recording method (BVP vs. ECG), age, health, measurement condition, artifacting procedures, and the concurrent-validity criteria used may have greater impact on ultra-short-term measurements than on longer recordings. Nonetheless, for healthy individuals, resting baselines as short as 1 min may be sufficient to measure HR, SDNN, and RMSSD as long as professionals carefully remove artifacts. The standardization of ultra-short-term measurement protocols and establishment of normative values for healthy non-athlete, optimal performance, and clinical populations remain important challenges to their use in place of conventional 5 min and 24 h values.

McNames and Aboy (130) compared 10 s to 10 min resting ECG recordings compared to 5 min recordings using archival data from PhysioNet. The strongest correlations were achieved with HF ms^2^, SDSD, and RMSSD. Salahuddin et al. (30) obtained 5 min of resting ECG data from 24 healthy students and noted that valid estimation of values from ultra-short-term recordings required differing lengths for different HRV variables: mean HR and RMSSD required 10 s; PNN50, HF (ms^2^ and nu), LF/HF, and LF nu required 20 s; LF ms^2^ required 30 s; VLF ms^2^ required 50 s; SDNN; the coefficient of variance required 60 s; HRV Index and TINN required 90 s; and the Stress Index required 100 s.

Similarly, Baek et al. (31) estimated 5 min resting PPG HRV values from 467 healthy volunteers with ultra-short-term recordings. HR required 10 s, HF ms^2^ required 20 s, RMSSD required 30 s, pNN50 required 60 s, LF (ms^2^ and nu), HF nu, and LF/HF ms^2^ required 90 s, SDNN required 240 s, and VLF ms^2^ required 270 s. These minimum periods also differed by age group.

When Nussinovitch et al. (126) compared 10 s and 1 min resting ECG recordings with 5 min recordings from 70 healthy volunteers, ultra-short-term RMSSD measurements achieved acceptable correlations, but SDNN did not achieve acceptable correlations with the longer short-term recordings.

Munoz et al. (129) measured SDNN and RMSSD in 3,387 adults and analyzed data using Pearson’s correlation coefficients, the Bland-Altman LoA method, and Cohen’s *d*. At 120 s, recordings achieved nearly perfect agreement with 240–300 s values (*r* = 0.956, bias = 0.406 for SDNN and 0.986, bias = 0.014 for RMSSD).

Shaffer et al. (128) recorded 5 min of resting ECG data from 38 healthy undergraduates and manually artifacted the IBIs. They correlated 10, 20, 30, 60, 90, 120, 180, and 240 s HRV metrics with 5 min metrics. The authors selected a conservative criterion of *r* = 0.90 to ensure that ultra-short-term values would account for at least 81% of the variability in 5 min values. A 10 s segment estimated mean HR. A 60 s segment measured SDNN, RMSSD, NN50, and pNN50. A 90 s segment calculated TINN, LF ms^2^, SD1, and SD2. A 120 s segment approximated HTI and DFA ɑ1. A 180 s segment computed LF nu, HF ms^2^, HF nu, LF/HF ms^2^, SampEn, DFA ɑ2, and DET. A 240 s segment assessed ShanEn. No UST measurement successfully estimated CD.

## Short-Term Measurement Norms

Short-term measurement norms are based on ~5 min of HRV data (Table 5). Because of their relative ease of recording, short-term measurements have been widely used and studied for many years, and appear to be the most commonly found source of published HRV data (11, 60). Short-term values are only appropriate when clients breathe at normal rates (~11–20 bpm). During resonance frequency biofeedback, the only relevant metrics are LF ms^2^ or peak frequency since breathing from 4.5 to 7.5 bpm concentrates HR oscillations around 0.1 Hz in the LF band.

**Table 5:** Short-term ECG norms.

Studies	Subjects	Spectral analysis	Breathing	Sample	Position	Metrics
Berkoff et al. (133)	145 elite athletes (87 M and 58 W) age 18–33	FFT	Free	2.5 min	Supine	SDNN, RMSSD, pNN50, LF (ms^2^ and nu), HF (power and nu), LF/HF (% and nu), and total power
Nunan et al. (17)	21,438 healthy adults (12,960 M and 8,474 W) age ≥ 40	AR and FFT	Free/paced	Varied	Varied	RR, SDNN, RMSSD, LF (ms^2^ and nu), HF (ms^2^ and nu), and LF/HF
Abhishekh et al. (105)	189 healthy adults (114 M and 75 W) age 16–60		Free	5 min	Supine	SDNN, RMSSD, LF (ms^2^ and nu), HF (ms^2^ and nu), LF/HF, and total power (ms^2^)
Seppälä et al. (134)	465 prepubertal children (239 B) and 226 G age 6–8	FFT	Free	5 min	Supine	RR, HR, SDNN, RMSSD, pNN50, HTI, TINN, LF (peak, ms^2^, %), HF (peak, ms^2^, %), LF/HF, SD1, SD2, SD1/SD2, SampEn, D_2_, DFA (α1 and α2) for 5^th^, 25^th^, 50^th^, 75^th^, and 95^th^ percentiles

*D_2_ (also CD), correlation dimension, which estimates the minimum number of variables required to construct a model of a studied system; DFA ɑ1, detrended fluctuation analysis, which describes short-term fluctuations; DFA ɑ2, detrended fluctuation analysis, which describes long-term fluctuations; ECG, electrocardiogram; HF ms^2^, absolute power of the high-frequency band; HF nu, relative power of the high-frequency band in normal units; HF peak, highest amplitude frequency in the HF band; HF%, HF power as a percentage of total power; HR, heart rate; HTI, HRV triangular index or integral of the density of the NN interval histogram divided by its height; LF ms^2^, absolute power of the low-frequency band; LF nu, relative power of the low-frequency band in normal units; LF peak, highest amplitude frequency in the LF band; LF%, LF power as a percentage of total power; LF/HF, ratio of LF-to-HF power; NN interval, time between adjacent normal heartbeats; nu, normal units calculated by dividing the absolute power for a specific frequency band by the summed absolute power of the LF and HF bands; pNN50, percentage of successive interbeat intervals that differ by more than 50 ms; RMSSD, root mean square of successive RR interval differences; RR interval, time between all adjacent heartbeats; SampEn, sample entropy, which measures signal regularity and complexity; SD1, Poincaré plot standard deviation perpendicular to the line of identity; SD2, Poincaré plot standard deviation along the line of identity; SD1/SD2, ratio of SD1 to SD2 that measures the unpredictability of the R–R time series and autonomic balance under appropriate monitoring conditions; SDNN, standard deviation of NN intervals; TINN, triangular interpolation of the RR interval histogram or baseline width of the RR interval histogram; total power, sum of power (ms^2^) in VLF, LF, and HF bands*.

Berkoff et al. (133) reported short-term norms from 145 elite track-and-field athletes (87 men and 58 women), 18–33 years, who were measured before the 2004 USA Olympic Trials. The investigators monitored the athletes in the supine position for 2.5 min using ECG after up to 5 min of rest to stabilize HR. These authors used the Fast Fourier transformation (FFT) method to perform power spectral analysis. They reported mean and standard deviation values by sex for the time-domain measures of SDNN, RMSSD, and pNN50 and the frequency-domain measures of LF (ms^2^ and nu), HF (ms^2^ and nu), LF/HF (LF/HF and LF/HF nu), and total power. Female athletes showed greater values for pNN50 and HF nu than male athletes. Male athletes showed greater values for LF nu and LF/HF ratio than female athletes. Type of sport (distance runners, field athletes, power athletes, sprinters, and strength athletes) did not affect HRV measures.

Normative data from short-term HRV studies published after the Task Force Report (11) were reviewed by Nunan et al. (17) (Table 6). The 44 selected studies meeting their criteria involved 21,438 healthy adult participants. This analysis included three large populations with a minimum age of 40 (135–137) which may explain their comparatively lower HRV values. The authors reported HRV values according to whether breathing was free or paced, sex, and spectral power analysis, autoregressive (AR) or FFT. They reported mean absolute and mean log-transformed values for mean RR, SDNN, RMSSD, LF (ms^2^ and nu), HF (ms^2^ and nu), and the LF/HF ratio. The selected studies showed greater agreement on time-domain measures (SDNN had the lowest coefficient of variation) than did frequency-domain measures (HF ms^2^ and log-transformed HF showed the largest variation). The FFT method resulted in lower LF power, greater HF power (ms^2^ and log-transformed), and greater LF/HF ratio than the AR method. PB resulted in higher values on all HRV indices except LF ms^2^, which was greatest during free breathing.

**Table 6:** Nunan et al. (17) short-term norms.

HRV measure	Mean (SD)	Range	Studies
IBI (ms)	926 (90)	785–1,160	30
SDNN (ms)	50 (16)	32–93	27
RMSSD (ms)	42 (15)	19–75	15
LF (ms^2^)	519 (291)	193–1,009	35
LF (nu)	52 (10)	30–65	29
HF (ms^2^)	657 (777)	83–3,630	36
HF (nu)	40 (10)	16–60	30
LF/HF (ms^2^)	2.8 (2.6)	1.1–11.6	25

*IBI, interbeat interval; SDNN, standard deviation of NN intervals; RMSSD, root mean square of successive RR interval differences; LF ms^2^, absolute power of the low-frequency band; LF nu, relative power of the low-frequency band in normal units; HF ms^2^, absolute power of the high-frequency band; HF nu, relative power of the high-frequency band in normal units; LF/HF, ratio of LF-to-HF power*.

*Reproduced with permission of John Wiley and Sons*.

More recently, Abhishekh et al. (105) studied 189 healthy participants (114 men and 75 women) who ranged from 16 to 60 years of age. They analyzed 5 min artifact-free supine ECG recordings obtained while participants breathed between 12 and 15 bpm. They reported SDNN and RMSSD time-domain measures, and LF (ms^2^ and nu), HF (ms^2^ and nu), the LF/HF ratio, and total power frequency-domain measures. The authors found a negative correlation of RMSSD, SDNN, and total power with age. While HF nu was negatively correlated with age, LF/HF ratio was positively correlated. These correlations suggested that sympathetic tone increases with age.

Seppälä et al. (134) monitored 465 prepubertal children (239 boys and 226 girls) 6–8 years of age. They obtained 1 and 5 min resting ECG recordings. They performed power spectral analysis using the FFT method. They reported mean RR interval and HR, SDNN, RMSSD, pNN50, HTI, and TINN HRV time-domain measures, LF (peak, ms^2^, and %), HF (peak, ms^2^, and %), LF/HF ms^2^, and SD1, SD2, SD1/SD2, SampEn, D2, and DFA (α1 and α2) non-linear measures. The authors reported 1 and 5 min reference values for these parameters for the 5th, 25th, 50th, 75th, and 95th percentiles and concluded that the same values could be used for both boys and girls since there were no gender differences. They argued that HRV parameters that reflect parasympathetic HR modulation (RMSSD, pNN50, HF ms^2^, and SD1) could be reliably measured using 1 min recordings. However, HTI, TINN, LF ms^2^, SD2, and relative LF and HF power, and SD1/SD2, require 5 min recordings due to the longer rhythms that comprise LF-band activity.

## Twenty-Four Hour Measurement Norms

Twenty-four-hour norms are obtained using ambulatory HRV monitoring (Table 7). The technology for recording and interpreting long-term “naturalistic” HR adjustments is rapidly advancing and the subject of another article in this issue (138). In the classic paper on the subject, The Task Force Report (11) reported 24 h norms for 144 healthy subjects that included cutoffs for moderately depressed and highly depressed HRV and for increased risk of mortality. The authors reported 24 h time-domain measures of SDNN, SDANN, RMSSD, and the HRV HTI, and supine 5 min frequency-domain measures for LF power (LF ms^2^ and nu), HF power (HF ms^2^ and nu), LF/HF power, and total power (ms^2^).

**Table 7:** Twenty-four-hour HRV norms.

Studies	Subjects	Metrics
Task Force Report (11)	274 healthy subjects (202 M and 72 F), age 40–69	24 h SDNN, SDANN, RMSSD, HTI and 5 min supine LF power (ms^2^ and nu), HF power (HF ms^2^ and HF nu), LF/HF power, and total power
Umetani et al. (32)	260 healthy subjects (122 M and 148 W), age 10–99	SDNN, SDANN, SDNNI, RMSSD, pNN50, and HR by decade
Beckers et al. (4)	276 healthy subjects (141 M and 135 W), age 18–71	SDNN, RMSSD, and pNN50, total power, LF (ms^2^ and %), HF (ms^2^ and %), and LF/HF ratio, and non-linear measures, 1/*f*, FD, DFA α1 and α2, CD, S, and LE
Bonnemeier et al. (139)	166 healthy subjects (85 M and 81 W), age 20–70	RMSSD, SDNN, SDNNI, SDANN, NN50, and HTI
Aeschbacher et al. (140)	2,079 subjects (972 M and 1,107 W), age 25–41	HR, SDNN, LF ms^2^ and HF ms^2^
Almeida-Santos et al. (106)	1,743 subjects (616 M and 1,127 W), age 40–100	SDNN, SDANN, SDNNI, RMSSD, and pNN50

*1/*f*, 1 divided by frequency slope, which characterizes the shape of the HRV frequency spectrum; CD, correlation dimension, which estimates the minimum number of variables required to construct a model of a studied system; DFA ɑ1, detrended fluctuation analysis, which describes short-term fluctuations; DFA ɑ2, detrended fluctuation analysis, which describes long-term fluctuations; ECG, electrocardiogram; FD, signal regularity; HF ms^2^, absolute power of the high-frequency band; HF nu, relative power of the high-frequency band in normal units; HF peak, highest amplitude frequency in the HF band; HF%, HF power as a percentage of total power; HR, heart rate; HTI, HRV triangular index or integral of the density of the NN interval histogram divided by its height; LE, Lyapunov exponent, which measures a non-linear system’s sensitive dependence on starting conditions; LF ms^2^, absolute power of the low-frequency band; LF nu, relative power of the low-frequency band in normal units; LF peak, highest amplitude frequency in the LF band; LF%, LF power as a percentage of total power; LF/HF, ratio of LF-to-HF power; NN interval, time between adjacent normal heartbeats; nu, normal units calculated by dividing the absolute power for a specific frequency band by the summed absolute power of the LF and HF bands; pNN50, percentage of successive interbeat intervals that differ by more than 50 ms; RMSSD, root mean square of successive RR interval differences; RR interval, time between all adjacent heartbeats; S, area of an ellipse fitting a Poincaré plot, which represents total HRV; SampEn, sample entropy, which measures signal regularity and complexity; SD1, Poincaré plot standard deviation perpendicular to the line of identity; SD2, Poincaré plot standard deviation along the line of identity; SD1/SD2, ratio of SD1 to SD2 that measures the unpredictability of the RR time series and autonomic balance under appropriate monitoring conditions; SDNN, standard deviation of NN intervals; TINN, triangular interpolation of the RR interval histogram or baseline width of the RR interval histogram; total power, sum of power (ms^2^) in ULF, VLF, LF, and HF bands*.

Umetani et al. (32) published 24 h norms for 260 healthy participants (112 men and 148 women) who ranged from 10 to 99 years old. The authors reported means, standard deviations, and 95% confidence intervals for 24 h HRV time-domain measures of SDNN, SDANN, SDNN index, RMSSD, and pNN50, and HR by decade. They analyzed the relationship between each HRV time-domain measure with HR and age, compared HR and HRV measures between decades and two-decade spans. They reported that several HRV time-domain indices declined with age. After age 65, subjects fell below cutoffs for increased threat of mortality. Before age 30, female subjects had lower HRV measurements than their male counterparts. This gender difference vanished after 50 years of age.

Beckers et al. (4) obtained 24 h ECG recordings of 276 healthy participants (141 men and 135 women) 18–71 years of age. They performed power spectral analysis using the FFT method and divided the 24 h recordings into daytime and nighttime. The authors reported day and night time-domain measures, SDNN, RMSSD, and pNN50, frequency-domain measures, total power (ms^2^), LF (ms^2^ and %), HF (ms^2^ and %), and LF/HF ratio, and non-linear measures, 1/frequency slope (1/*f*), fractal dimension (FD), DFA α1 and α2, CD, % of CD difference, S value, and Lyapunov exponent. Both linear and non-linear metrics decreased with age. The authors found that non-linear values were higher at night, did not differ by sex, and decreased with age.

Bonnemeier et al. (139) recorded 24 h ECG for 166 healthy volunteers (85 men and 81 women) aged 20–70. They obtained hourly and 24 h RMSSD, SDNN. SDNNI, SDANN, NN50, and HTI values. All 24 h HRV values declined with age. The attenuation of HRV parameters with age mainly occurred during nighttime. The largest decrease occurred during the second and third decades. Following this drop, the decline was gradual. SDNNI, NN50, and RMMSD correlated most strongly with aging. Mean 24 h RR interval, SDNN, SDNNI (SD for all 5 min intervals) and SDANN were significantly higher in men. Gender differences diminished with age.

Aeschbacher et al. (140) recorded 24 h ambulatory ECGs and assessed the lifestyles of 2,079 subjects (972 men and 1,107) aged 25–41. They obtained HR, SDNN, and LF and HF power. SDNN was 160 ± 40 (men) and 147 ± 36 (women). LF power was 1,337 ms^2^ (men) and 884 ms^2^ (women). HF power was 289 ms^2^ (men) and 274 ms^2^ (women). The authors reported that only a minority of their sample had healthy lifestyles and that lifestyle scores were associated with 24 h SDNN values.

Almeida-Santos et al. (106) obtained 22–24 h ambulatory ECGs from 1,743 participants (616 men and 1,127 women) aged 40–100. While their sample included comorbidities like dyslipidemia and hypertension, they were capable of performing the activities of daily living. The authors calculated HRV time-domain measures of SDNN, SDANN, SDNNI, RMSSD, and pNN50. HRV linearly declined with age. SDNN, SDANN, SDNNI, RMSSD, and PNN50 were higher in men than women. RMSSD and pNN50 showed a U-shaped pattern with aging, decreasing from 40 to 60 and then increasing from 70. The authors concluded that global autonomic regulation decreases linearly with aging and is lower in men, diabetics, and obese individuals.

## Assessment in Clinical and Optimal Performance Interventions

The selection of HRV time-domain, frequency-domain, and non-linear measurements and norms to assess progress in clinical and optimal performance interventions should be informed by peer-reviewed studies. Professionals training specialized populations (e.g., chronic pain patients) might supplement published norms for the general population with values from their own clients. The rigorous data reporting guidelines proposed by Laborde et al. (93) could guide their efforts to publish their norms to remedy gaps in the literature. The metrics most strongly correlated with clinical improvement and athlete performance gains in these reports could be incorporated in pretreatment/posttreatment, within-session, and across-session assessment. While a full treatment of HRV variables in relation to the HRV biofeedback intervention is beyond the scope of this article, we will briefly touch on the issues that seem to us to be the key ones (141).

In addition to the primary literature, the Association for Applied Psychophysiology and Biofeedback has published two references that identify metrics associated with clinical and optimal performance outcomes, *Evidence-Based Practice in Biofeedback and Neurofeedback* (3rd ed.) and *Foundations of Heart Rate Variability Biofeedback: A Book of Readings* (142, 143). Further, readers might consult Gevirtz, Lehrer, and Schwartz’s excellent chapter on Cardiorespiratory Biofeedback in Schwartz and Andrasik’s (Eds.) *Biofeedback: A Practitioner*’*s Guide* (4th ed.).

## Pretreatment/Posttreatment Assessment

Twenty-four-hour HRV monitoring before and after a series of HRV biofeedback training sessions provides the most valid measurements of ULF, VLF, total power, and LF/HF-domain indices (12). Moreover, 24 h time-domain measurements like SDNN achieve prognostic power that ultra-short-term and short-term measurements cannot. Successful HRV biofeedback should result in increased power in all individual frequency bands, total power, and LF/HF ratio, and relevant time-domain and non-linear values.

Where 24 h HRV assessment is not feasible, short-term (~5 min) resting measurements without feedback or pacing, and while breathing at normal rates can help evaluate physiological change. Successful HRV biofeedback should increase LnHF (which may index vagal tone under controlled conditions), RSA, and possibly LF and total power, and relevant time-domain and non-linear values. Autonomic (finger temperature and skin conductance/potential) and respiratory (end-tidal CO_2_ and respiration depth, rate, and rhythmicity) indices can complement HRV measurements. Successful HRV biofeedback may increase finger temperature, decrease skin conductance/potential, increase end-tidal CO_2_ to between 35 and 45 torr, increase respiration depth, slow respiration rate below 16 bpm, and increase rhythmicity in respirometer and HR waveforms (144).

## Within- and Across-Session Assessment

Short-term resting HRV, autonomic, and respiratory measurements without feedback or pacing, and while breathing at normal rates can be obtained during pre- and posttraining baselines for within-session assessment or across the pretraining baselines of successive sessions. For both within- and across-session assessment, successful HRV biofeedback training should result in the same pattern of physiological change as described the previous section on short-term resting pretreatment/posttreatment assessment. While increased VLF power in 24 h HRV assessment is consistent with improved health, this change during short-term assessment may indicate training difficulty, *vagal withdrawal*, due to excessive effort (56). Where short-term assessment does not involve physical exercise or stress trials, the LF/HF ratio may not index of autonomic balance since there will be no significant SNS activation to measure (12).

## Assessment during HRV Biofeedback Trials

During HRV biofeedback training, adults may be instructed to engage in paced abdominal breathing between 4.5 and 7.5 bpm guided by a real-time display of instantaneous HR and respiration. As clients’ breathing approaches their *resonance frequency*, the rate that most strongly stimulates their baroreceptor reflex, RSA will increase (141). Since respiration rate helps to determine the peak HRV frequency (the frequency with the highest amplitude), successful training should produce a lower peak frequency and greater LF power than a resting baseline obtained when clients breathe from 12 to 15 bpm. PB at 6 bpm should result in a spectral peak at 0.1 Hz, while breathing at 7.5 bpm should create a peak at 0.125 Hz. Both 6 and 7.5 bpm rates will also increase power in the LF, which ranges from 0.04 to 0.15 Hz.

## Summary

Autonomic efferent neurons and circulating hormones modulate SA node initiation of heartbeats. The interdependent regulatory systems that generate the complex variability of a healthy heart operate over different time scales to achieve homeostasis and optimal performance. Circadian oscillations in circadian variations in core body temperature, metabolism, sleep–wake cycles, and the renin–angiotensin system contribute to 24 h HRV measurements. The complex dynamic relationship between the sympathetic and parasympathetic branches, and homeostatic regulation of HR *via* respiration and the baroreceptor reflex are responsible for short-term and ultra-short-term HRV measurements. Since slower regulatory mechanisms contribute to HRV metrics recorded over longer measurement periods, 24 h, short-term, and ultra-short-term values are not interchangeable.

Clinicians and researchers measure HRV using time-domain, frequency-domain, and non-linear indices. Time-domain values measure how much HRV was observed during the monitoring period. Recording period length strongly influences time-domain values. Shorter epochs are associated with smaller values and poorly estimate 24 h values (17). For example, where 24 h SDNN values predict future heart attack risk, 5 min SDNN values do not (12).

Frequency-domain values calculate absolute or relative signal power within the ULF, VLF, LF, and HF bands. Recording period length limits HRV frequency-band measurement. Minimum recommended periods include: ULF (24 h), VLF (5 min, 24 h preferred), LF (2 min), and HF (1 min). Again, short-term epochs (~5 min) lack the prognostic power of 24 h measurements for morbidity and mortality.

Non-linear indices measure the unpredictability and complexity of a series of IBIs. The relationship between non-linear measurements and illness is complex. While stressors and disease lower some non-linear indices, in cases like myocardial infarction, higher non-linear HRV predicts a greater risk of mortality.

The expanding literature on ultra-short-term, short-term, and 24 h HRV norms requires careful interpretation. Due to the lack of standardization of ultra-short-term measurement protocols, concurrent validity criteria, and normative values for healthy non-athlete, optimal performance, and clinical populations, clinicians should not use ultra-short-term interchangeably with 5 min and 24 h values.

Short-term measurement norms can contribute to assessment before, during, and after HRV biofeedback training for both clinical and optimal performance. Since short-term measurement norm studies vary in detection method (ECG or PPG), frequency-band cutoffs, power spectral analysis method (AR or FFT), position (sitting upright or lying supine), respiration rate, and breathing pacing (paced or free breathing) and subject sex, age, and aerobic fitness, the selection of appropriate norms is crucial. Likewise, 24 h HRV norms can guide HRV biofeedback training for clinical and optimal performance. As with short-term measurement norms, frequency-band cutoffs, power spectral analysis method (AR or FFT), and subject sex, age, and aerobic fitness can help to determine the selection of reference values.

The selection of HRV time-domain, frequency-domain, and non-linear metrics to assess progress in clinical and optimal performance interventions can be guided by peer-reviewed studies and supplemented by values from specialized populations. The HRV metrics most strongly correlated with clinical improvement and athlete performance gains in these reports might be incorporated in pretreatment/posttreatment, within-session, and across-session assessment. Finally, LF-band power and RSA will increase during successful HRV biofeedback trials due to PB in the 4.5–7.5 bpm range.

## Author Contributions

FS reviewed the literature, revised JG’s article outline, wrote the initial abstract, manuscript, and table drafts, and revised the second drafts following feedback from JG. JG reviewed the literature, proposed an article outline, created and maintained an EndNote database, contributed sections of the initial drafts, and made editorial suggestions for the second drafts. Both FS and JG discussed the conceptual issues and themes for the review article.

## Conflict of Interest Statement

The authors declare that the research was conducted in the absence of any commercial or financial relationships that could be construed as a potential conflict of interest.

## References

[1] McCraty R Shaffer F. Heart rate variability: new perspectives on physiological mechanisms, assessment of self-regulatory capacity, and health risk. Glob Adv Health Med (2015) 4:46–61. 10.7453/gahmj.2014.073 25694852 PMC4311559

[2] Gevirtz RN Lehrer PM Schwartz MS Cardiorespiratory biofeedback. 4th ed In: Schwartz MS Andrasik F, editors. Biofeedback: A Practitioner’s Guide. New York: The Guilford Press (2016). p. 196–213.

[3] Goldberger AL. Is the normal heartbeat chaotic or homeostatic? News Physiol Sci (1991) 6:87–91. 11537649 10.1152/physiologyonline.1991.6.2.87

[4] Beckers F Verheyden B Aubert AE. Aging and nonlinear heart rate control in a healthy population. Am J Physiol Heart Circ Physiol (2006) 290:H2560–70. 10.1152/ajpheart.00903.2005 16373585

[5] Vaillancourt DE Newell KM. Changing complexity in human behavior and physiology through aging and disease. Neurobiol Aging (2002) 23:1–11. 10.1016/S0197-4580(01)00247-0 11755010

[6] Stein PK Domitrovich PP Hui N Rautaharju P Gottfdiener J J Cardiovasc Electrophysiol (2005) 16:954–9. 10.1111/j.1540-8167.2005.40788.x 16174015

[7] Lane RD Reiman EM Ahem GL Thayer JF Activity in medial prefrontal cortex correlates with vagal component of heart rate variability during emotion. Brain Cognit (2001) 47:97–100.

[8] Tarvainen MP Lipponen J Niskanen JP Ranta-Aho P Kubios HRV Version 3 – User’s Guide. Kuopio: University of Eastern Finland (2017).

[9] Kuusela T Methodological aspects of heart rate variability analysis. In: Kamath MV Watanabe MA Upton ARM, editors. Heart Rate Variability (HRV) Signal Analysis. Boca Raton, FL: CRC Press (2013). p. 9–42.

[10] Kleiger RE Stein PK Bigger JT Jr. Heart rate variability: measurement and clinical utility. Ann Noninvasive Electrocardiol (2005) 10:88–101. 10.1111/j.1542-474X.2005.10101.x 15649244 PMC6932537

[11] Task Force Report. Heart rate variability: standards of measurement, physiological interpretation, and clinical use. Circulation (1996) 93:1043–65. 10.1161/01.CIR.93.5.1043 8598068

[12] Shaffer F McCraty R Zerr CL A healthy heart is not a metronome: an integrative review of the heart’s anatomy and heart rate variability. Front Psychol (2014) 5:1040 10.3389/fpsyg.2014.01040 25324790 PMC4179748

[13] Stein PK Reddy A Non-linear heart rate variability and risk stratification in cardiovascular disease. Indian Pacing Electrophysiol J (2005) 5:210–20. 16943869 PMC1431594

[14] Karemaker JM Counterpoint: respiratory sinus arrhythmia is due to the baroreflex mechanism. J Appl Psychol (2009) 106:1742–3. 10.1152/japplphysiol.91107.2008a 19414625

[15] Tortora GJ Derrickson BH Principles of Anatomy and Physiology. 15th ed New York: John Wiley and Sons, Inc (2017).

[16] Olshansky B Sabbah HN Hauptman PJ Colucci WS Parasympathetic nervous system and heart failure: pathophysiology and potential implications for therapy. Circulation (2008) 118:863–71. 10.1161/CIRCULATIONAHA.107.760405 18711023

[17] Nunan D Sandercock GRH Brodie DA. A quantitative systematic review of normal values for short-term heart rate variability in healthy adults. Pacing Clin Electrophysiol (2010) 33:1407–17. 10.1111/j.1540-8159.2010.02841.x 20663071

[18] Gellhorn E Autonomic Imbalance and the Hypthalamus: Implications for Physiology, Medicine, Psychology, and Neuropsychiatry. London: Oxford University Press (1957).

[19] Nada T Nomura M Iga A Kawaguchi R Ochi Y Saito K Autonomic nervous function in patients with peptic ulcer studied by spectral analysis of heart rate variability. J Med (2001) 32:333–47. 11958279

[20] Ballard RD Sleep, respiratory physiology, and nocturnal asthma. Chronobiol Int (1999) 5:565–80. 10.3109/07420529908998729 10513883

[21] Billman GE The LF/HF ratio does not accurately measure cardiac sympatho-vagal balance. Front Physiol (2013) 4:26 10.3389/fphys.2013.00026 23431279 PMC3576706

[22] Billman GE Huikuri HV Sacha J Trimmel K An introduction to heart rate variability: methodological considerations and clinical applications. Front Physiol (2015) 6:55 10.3389/fphys.2015.00055 25762937 PMC4340167

[23] Eckberg DL Sleight P Human Baroreflexes in Health and Disease. Oxford: Clarendon Press (1992).

[24] Gevirtz RN Lehrer P Resonant frequency heart rate biofeedback. 3rd ed In: Schwartz MS Andrasik F, editors. Biofeedback: A Practitioner’s Guide. New York: The Guilford Press (2003). p. 245–50.

[25] Lehrer PM Vaschillo E The future of heart rate variability biofeedback. Biofeedback (2008) 36:11–4.

[26] Vaschillo E Lehrer P Rishe N Konstantinov M. Heart rate variability biofeedback as a method for assessing baroreflex function: a preliminary study of resonance in the cardiovascular system. Appl Psychophysiol Biofeedback (2002) 27:1–27. 10.1023/A:1014587304314 12001882

[27] Bigger JT Jr Albrecht P Steinman RC Rolnitzky LM Fleiss JL Cohen RJ Comparison of time- and frequency domain-based measures of cardiac parasympathetic activity in Holter recordings after myocardial infarction. Am J Cardiol (1989) 64:536–8. 10.1016/0002-9149(89)90436-0 2773799

[28] Fei L Copie X Malik M Camm AJ. Short- and long-term assessment of heart rate variability for risk stratification after acute myocardial infarction. Am J Cardiol (1996) 77:681–4. 10.1016/S0002-9149(97)89199-0 8651116

[29] Nolan J Batin PD Andrews R Lindsay SJ Brooksby P Mullen M Prospective study of heart rate variability and mortality in chronic heart failure: results of the United Kingdom heart failure evaluation and assessment of risk trial (UK-heart). Circulation (1998) 98:1510–6. 10.1161/01.CIR.98.15.1510 9769304

[30] Salahuddin L Cho J Jeong MG Kim D. Ultra short term analysis of heart rate variability for monitoring mental stress in mobile settings. Conf Proc IEEE Eng Med Biol Soc (2007) 2007:4656–9. 18003044 10.1109/IEMBS.2007.4353378

[31] Baek HJ Cho CH Cho J Woo JM. Reliability of ultra-short-term analysis as a surrogate of standard 5-min analysis of heart rate variability. Telemed J E Health (2015) 21:404–14. 10.1089/tmj.2014.0104 25807067

[32] Umetani K Singer DH McCraty R Atkinson M. Twenty-four hour time domain heart rate variability and heart rate: relations to age and gender over nine decades. J Am Coll Cardiol (1998) 31:593–601. 10.1016/S0735-1097(97)00554-8 9502641

[33] Grant CC van Rensburg DC Strydom N Viljoen M Importance of tachogram length and period of recording during noninvasive investigation of the autonomic nervous system. Ann Noninvasive Electrocardiol (2011) 16:131–9. 10.1111/j.1542-474X.2011.00422.x 21496163 PMC6932447

[34] Kleiger RE Miller JP Bigger JT Jr Moss AJ. Decreased heart rate variability and its association with increased mortality after acute myocardial infarction. Am J Cardiol (1987) 59:256–62. 10.1016/0002-9149(87)90795-8 3812275

[35] Otzenberger H Gronfier C Simon C Charloux A Ehrhart J Piquard F Dynamic heart rate variability: a tool for exploring sympathovagal balance continuously during sleep in men. Am J Physiol (1998) 275(3 Pt 2):H946–50. 9724299 10.1152/ajpheart.1998.275.3.H946

[36] Esco MR Flatt AA. Ultra-short-term heart rate variability indexes at rest and post-exercise in athletes: evaluating the agreement with accepted recommendations. J Sports Sci Med (2014) 13:535–41. 25177179 PMC4126289

[37] Ciccone AB Siedlik JA Wecht JM Deckert JA Nguyen ND Weir JP. Reminder: RMSSD and SD1 are identical heart rate variability metrics. Muscle Nerve (2017). 10.1002/mus.25573 28073153

[38] Burr RL Motzer SA Chen W Cowan MJ Shulman RJ Heitkemper MM. Heart rate variability and 24-hour minimum heart rate. Biol Res Nurs (2006) 7(4):256–67. 10.1177/1099800405285268 16581896

[39] Schipke JD Arnold G Pelzer M Effect of respiration rate on short-term heart rate variability. J Clin Basic Cardiol (1999) 2:92–5.

[40] Pentillä J Helminen A Jarti T Kuusela T Huikuri HV Tulppo MP Time domain, geometrical and frequency domain analysis of cardiac vagal outflow: effects of various respiratory patterns. Clin Phys (2001) 21:365–76. 10.1046/j.1365-2281.2001.00337.x 11380537

[41] Hill LK Siebenbrock A Are all measures created equal? Heart rate variability and respiration – biomed 2009. Biomed Sci Instrum (2009) 45:71–6. 19369742

[42] DeGiorgio CM Miller P Meymandi S Chin A Epps J Gordon S RMSSD, a measure of vagus-mediated heart rate variability, is associated with risk factors for SUDEP: the SUDEP-7 inventory. Epilepsy Behav (2010) 19(78–81):78–81. 10.1016/j.yebeh.2010.06.011 20667792 PMC2943000

[43] Jovic A Bogunovic N. Electrocardiogram analysis using a combination of statistical, geometric, and nonlinear heart rate variability features. Artif Intell Med (2011) 51:175–86. 10.1016/j.artmed.2010.09.005 20980134

[44] Bigger JT Jr Fleiss JL Steinman RC Rolnitzky LM Kleiger RE Rottman JN. Frequency domain measures of heart period variability and mortality after myocardial infarction. Circulation (1992) 85:164–71. 10.1161/01.CIR.85.1.164 1728446

[45] Bonaduce D Petretta M Morgano G Villari B Bianchi V Conforti G Left ventricular remodelling in the year after myocardial infarction: an echocardiographic, haemodynamic, and radionuclide angiographic study. Coron Artery Dis (1994) 5:155–62. 10.1097/00019501-199402000-00009 8180745

[46] Stampfer HG The relationship between psychiatric illness and the circadian pattern of heart rate. Aust NZ J Psychiatry (1998) 32:187–98. 10.3109/00048679809062728 9588297

[47] Stampfer HG Dimmitt SB Variations in circadian heart rate in psychiatric disorders: theoretical and practical implications. Chronophysiol Ther (2013) 3:41–50. 10.2147/CPT.S43623

[48] Tsuji H Venditti FJ Jr Manders ES Evans JC Larson MG Feldman CL Reduced heart rate variability and mortality risk in an elderly cohort. The Framingham Heart Study. Circulation (1994) 90:878–83. 10.1161/01.CIR.90.2.878 8044959

[49] Tsuji H Larson MG Venditti FJ Jr Manders ES Evans JC Feldman CL Impact of reduced heart rate variability on risk for cardiac events. The Framingham Heart Study. Circulation (1996) 94:2850–5. 10.1161/01.CIR.94.11.2850 8941112

[50] Hadase M Azuma A Zen K Asada S Kawasaki T Kamitani T Very low frequency power of heart rate variability is a powerful predictor of clinical prognosis in patients with congestive heart failure. Circ J (2004) 68:343–7. 10.1253/circj.68.343 15056832

[51] Schmidt H Müller-Werdan U Hoffmann T Francis DP Piepoli MF Rauchhaus M Autonomic dysfunction predicts mortality in patients with multiple organ dysfunction syndrome of different age groups. Crit Care Med (2005) 33:1994–2002. 10.1097/01.CCM.0000178181.91250.99 16148471

[52] Shah AJ Lampert R Goldberg J Veledar E Bremner JD Vaccarino V. Posttraumatic stress disorder and impaired autonomic modulation in male twins. Biol Psychiatry (2013) 73:1103–10. 10.1016/j.biopsych.2013.01.019 23434412 PMC3648627

[53] Carney RM Freedland KE Stein PK Miller GE Steinmeyer B Rich MW Heart rate variability and markers of inflammation and coagulation in depressed patients with coronary heart disease. J Psychosom Res (2007) 62:463–7. 10.1016/j.jpsychores.2006.12.004 17383498 PMC1924882

[54] Lampert R Bremner JD Su S Miller A Lee F Cheema F Decreased heart rate variability is associated with higher levels of inflammation in middle-aged men. Am Heart J (2008) 156:759.e1–7. 10.1016/j.ahj.2008.07.009 18926158 PMC2587932

[55] Theorell T Liljeholm-Johansson Y Björk H Ericson M Saliva testosterone and heart rate variability in the professional symphony orchestra after “public faintings” of an orchestra member. Psychoneuroendocrinology (2007) 32:660–8. 10.1016/j.psyneuen.2007.04.006 17560732

[56] Bernardi L Valle F Coco M Calciati A Sleight P. Physical activity influences heart rate variability and very-low-frequency components in Holter electrocardiograms. Cardiovasc Res (1996) 32:234–7. 10.1016/0008-6363(96)00081-8 8796109

[57] Akselrod S Gordon D Ubel FA Shannon DC Barger AC Cohen RJ. Power spectrum analysis of heart rate fluctuation: a quantitative probe of beat-to-beat cardiovascular control. Science (1981) 213:220–2. 10.1126/science.6166045 6166045

[58] Claydon VE Krassioukov AV. Clinical correlates of frequency analyses of cardiovascular control after spinal cord injury. Am J Physiol Heart Circ Physiol (2008) 294:H668–78. 10.1152/ajpheart.00869.2007 18024546

[59] Taylor JA Carr DL Myers CW Eckberg DL. Mechanisms underlying very-low-frequency RR-interval oscillations in humans. Circulation (1998) 98:547–55. 10.1161/01.CIR.98.6.547 9714112

[60] Berntson GG Bigger JT Jr Eckberg DL Grossman P Kaufmann PG Malik M Heart rate variability: origins, methods, and interpretive caveats. Psychophysiology (1997) 34:623–48. 10.1111/j.1469-8986.1997.tb02140.x 9401419

[61] Armour JA Neurocardiology: Anatomical and Functional Principles. Boulder Creek, CA: Institute of HeartMath (2003).

[62] Kember GC Fenton GA Collier K Armour JA. Aperiodic stochastic resonance in a hysteretic population of cardiac neurons. Phys Rev E Stat Phys Plasmas Fluids Relat Interdiscip Topics (2000) 61:1816–24. 10.1103/PhysRevE.61.1816 11046466

[63] Kember GC Fenton GA Armour JA Kalyaniwalla N. Competition model for aperiodic stochastic resonance in a Fitzhugh-Nagumo model of cardiac sensory neurons. Phys Rev E Stat Nonlin Soft Matter Phys (2001) 63(4 Pt 1):041911. 10.1103/PhysRevE.63.041911 11308881

[64] Berntson GG Cacioppo JT Grossman P Wither vagal tone. Biol Psychol (2007) 74:295–300. 10.1016/j.biopsycho.2006.08.006 17046142

[65] Lehrer PM Biofeedback training to increase heart rate variability. In: Lehrer PM Woolfolk RL Sime WE, editors. Principles and Practice of Stress Management. New York, NY: The Guilford Press (2007). p. 227–48.

[66] Reyes del Paso GA Langewitz W Mulder LJM Van Roon A Duschek S. The utility of low frequency heart rate variability as an index of sympathetic cardiac tone: a review with emphasis on a reanalysis of previous studies. Psychophysiology (2013) 50:477–87. 10.1111/psyp.12027 23445494

[67] Goldstein DS Bentho O Park MY Sharabi Y. Low-frequency power of heart rate variability is not a measure of cardiac sympathetic tone but may be a measure of modulation of cardiac autonomic outflows by baroreflexes. Exp Physiol (2011) 96:1255–61. 10.1113/expphysiol.2010.056259 21890520 PMC3224799

[68] Ahmed AK Harness JB Mearns AJ Respiratory control of heart rate. Eur J Appl Physiol (1982) 50:95–104. 10.1007/BF00952248

[69] Lehrer PM Vaschillo E Vaschillo B Lu SE Eckberg DL Edelberg R Heart rate variability biofeedback increases baroreflex gain and peak expiratory flow. Psychosom Med (2003) 65:796–805. 10.1097/01.PSY.0000089200.81962.19 14508023

[70] Tiller WA McCraty R Atkinson M. Cardiac coherence: a new, noninvasive measure of autonomic nervous system order. Altern Ther Health Med (1996) 2:52–65. 8795873

[71] Brown TE Beightol LA Koh J Eckberg DL. Important influence of respiration on human R-R interval power spectra is largely ignored. J Appl Physiol (1985) (1993) 75:2310–7. 8307890 10.1152/jappl.1993.75.5.2310

[72] Quintana DS Elstad M Kaufmann T Brandt CL Haatveit B Haram M Resting-state high-frequency heart rate variability is related to respiratory frequency in individuals with severe mental illness but not healthy controls. Sci Rep (2016) 6:37212 10.1038/srep37212 27853244 PMC5112550

[73] Grossman P Taylor EW. Toward understanding respiratory sinus arrhythmia: relations to cardiac vagal tone, evolution and biobehavioral functions. Biol Psychol (2007) 74:263–85. 10.1016/j.biopsycho.2005.11.014 17081672

[74] Eckberg DL Eckberg MJ. Human sinus node responses to repetitive, ramped carotid baroreceptor stimuli. Am J Physiol (1982) 242:H638–44. 7065276 10.1152/ajpheart.1982.242.4.H638

[75] Thayer JF Yamamoto SS Brosschot JF. The relationship of autonomic imbalance, heart rate variability and cardiovascular disease risk factors. Int J Cardiol (2010) 141:122–31. 10.1016/j.ijcard.2009.09.543 19910061

[76] Grossman P Comment on heart rate variability and cardiac vagal tone in psychophysiological research – recommendations for experiment planning, data analysis, and data reporting. Front Psychol (2017) 8:213 10.3389/fpsyg.2017.00213 28265249 PMC5316555

[77] Egizio VB Eddy M Robinson M Jennings JR. Efficient and cost-effective estimation of the influence of respiratory variables on respiratory sinus arrhythmia. Psychophysiology (2011) 48:488–94. 10.1111/j.1469-8986.2010.01086.x 20718933 PMC2990812

[78] Pagani M Lombardi F Guzzetti S Sandrone G Rimoldi O Malfatto G Power spectral density of heart rate variability as an index of sympatho-vagal interaction in normal and hypertensive subjects. J Hypertens Suppl (1984) 2:S383–5. 6599685

[79] Pagani M Lombardi F Guzzetti S Rimoldi O Furlan RA Pizzinelli P Power spectral analysis of heart rate and arterial pressure variabilities as a marker of sympatho-vagal interaction in man and conscious dog. Circ Res (1986) 59:178–93. 10.1161/01.RES.59.2.178 2874900

[80] Eckberg DL Human sinus arrhythmia as an index of vagal outflow. J Appl Physiol Respir Environ Exerc Physiol (1983) 54:961–6. 6853303 10.1152/jappl.1983.54.4.961

[81] Schrödinger E What is Life? The Physical Aspect of the Living Cell. Cambridge: Cambridge University Press (1944).

[82] Stein PK Reddy A Non-linear heart rate variability and risk stratification in cardiovascular disease. Indian Pacing Electrophysiol J (2005) 5:210–20. 16943869 PMC1431594

[83] Behbahani S Dabanloo NJ Nasrabadi AM Ictal heart rate variability assessment with focus on secondary generalized and complex partial epileptic seizures. Adv Biores (2012) 4:50–8.

[84] Zerr C Kane A Vodopest T Allen J Hannan J Cangelosi A The nonlinear index SD1 predicts diastolic blood pressure and HRV time and frequency domain measurements in healthy undergraduates [Abstract]. Appl Psychophysiol Biofeedback (2015) 40:134 10.1007/s10484-015-9282-0

[85] Zerr C Kane A Vodopest T Allen J Hannan J Fabbri M Does inhalation-to-exhalation ratio matter in heart rate variability biofeedback? [Abstract]. Appl Psychophysiol Biofeedback (2015) 40:135 10.1007/s10484-015-9282-0

[86] Brennan M Palaniswami M Kamen P. Do existing measures of Poincaré plot geometry reflect nonlinear features of heart rate variability? IEEE Trans Biomed Eng (2001) 48:1342–7. 10.1109/10.959330 11686633

[87] Brennan M Palaniswami M Kamen P. Poincaré plot interpretation using a physiological model of HRV based on a network of oscillators. Am J Physiol Heart Circ Physiol (2002) 283:H1873–86. 10.1152/ajpheart.00405.2000 12384465

[88] Tulppo MP Mäkikallio TH Takala TE Seppänen T Huikuri HV. Quantitative beat-to-beat analysis of heart rate dynamics during exercise. Am J Physiol (1996) 271:H244–52. 8760181 10.1152/ajpheart.1996.271.1.H244

[89] Tulppo MP Mäkikallio TH Seppänen T Laukkanen RT Huikuri HV. Vagal modulation of heart rate during exercise: effects of age and physical fitness. Am J Physiol (1998) 274(2 Pt 2):H424–9. 9486244 10.1152/ajpheart.1998.274.2.H424

[90] Guzik P Piskorski J Krauze T Schneider R Wesseling KH Wykretowicz A Correlations between the Poincaré plot and conventional heart rate variability parameters assessed during paced breathing. J Physiol Sci (2007) 57:63–71. 10.2170/physiolsci.RP005506 17266795

[91] Beckers F Ramaekers D Aubert AE Approximate entropy of heart rate variability: validation of methods and application in heart failure. Cardiovasc Eng (2001) 1:177–82. 10.1023/A:1015212328405

[92] Lippman N Stein KM Lerman BB. Comparison of methods for removal of ectopy in measurement of heart rate variability. Am J Physiol (1994) 267(1 Pt 2):H411–8. 7519408 10.1152/ajpheart.1994.267.1.H411

[93] Laborde S Mosley E Thayer JF. Heart rate variability and cardiac vagal tone in psychophysiological research – recommendations for experiment planning, data analysis, and data reporting. Front Psychol (2017) 8:213. 10.3389/fpsyg.2017.00213 28265249 PMC5316555

[94] Saul JP Albrecht P Berger RD Cohen RJ Analysis of long-term heart rate variability: methods, 1/f scaling and implications. Comput Cardiol (1988) 14:419–22. 11542156

[95] Jeyhani V Mahdiani S Peltokangas M Vehkaja A. Comparison of HRV parameters derived from photoplethysmography and electrocardiography signals. Conf Proc IEEE Eng Med Biol Soc (2015) 2015:5952–5. 10.1109/EMBC.2015.7319747 26737647

[96] Merri M Farden DC Mottley JG Titlebaum EL. Sampling frequency of the electrocardiogram for spectral analysis of the heart rate variability. IEEE Trans Biomed Eng (1990) 37:99–106. 10.1109/10.43621 2303276

[97] Peltola MA. Role of editing of R-R intervals in the analysis of heart rate variability. Front Physiol (2012) 23:148. 10.3389/fphys.2012.00148 22654764 PMC3358711

[98] Berntson GG Stowell JR ECG artifacts and heart period variability: don’t miss a beat! Psychophysiology (1998) 35:127–32. 10.1111/1469-8986.3510127 9499713

[99] Shaffer F Combatalade DC Don’t add or miss a beat: a guide to cleaner heart rate variability recordings. Biofeedback (2013) 41:121–30. 10.5298/1081-5937-41.3.04

[100] Hirsch JA Bishop B. Respiratory sinus arrhythmia in humans: how breathing pattern modulates heart rate. Am J Physiol (1981) 241:H620–9. 7315987 10.1152/ajpheart.1981.241.4.H620

[101] Meehan Z Muesenfechter N Gravett N Watson T Smith A Shearman S Breathing effort may not reduce heart rate variability when respiration rate is controlled [Abstract]. Appl Psychophysiol Biofeedback (Forthcoming).

[102] Meehan Z Muesenfechter N Gravett N Watson T Smith A Shearman S A 1:2 inhalation-to-exhalation ratio does not increase heart rate variability during 6-bpm breathing [Abstract]. Appl Psychophysiol Biofeedback (Forthcoming).

[103] Lin IM Tai L Fan SY. Breathing at a rate of 5.5 breaths per minute with equal inhalation-to-exhalation ratio increases heart rate variability. Int J Psychophysiol (2014) 91:206–11. 10.1016/j.ijpsycho.2013.12.006 24380741

[104] Bonnemeier H Richardt G Potratz J Wiegand UK Brandes A Kluge N Circadian profile of cardiac autonomic nervous modulation in healthy subjects: differing effects of aging and gender on heart rate variability. J Cardiovasc Electrophysiol (2003) 14:791–9. 10.1046/j.1540-8167.2003.03078.x 12890036

[105] Abhishekh HA Nisarga P Kisan R Meghana A Chandran S Raju T Influence of age and gender on autonomic regulation of heart. J Clin Monit Comput (2013) 27:259–64. 10.1007/s10877-012-9424-3 23297094

[106] Almeida-Santos MA Barreto-Filho JA Oliveira JL Reis FP da Cunha Oliveira CC Sousa AC. Aging, heart rate variability and patterns of autonomic regulation of the heart. Arch Gerontol Geriatr (2016) 63:1–8. 10.1016/j.archger.2015.11.011 26791165

[107] Koenig J Thayer JF. Sex differences in healthy human heart rate variability: a meta-analysis. Neurosci Biobehav Rev (2016) 64:288–310. 10.1016/j.neubiorev.2016.03.007 26964804

[108] Zhang D Shen X Qi X. Resting heart rate and all-cause and cardiovascular mortality in the general population: a meta-analysis. CMAJ (2016) 188:E53–63. 10.1503/cmaj.150535 26598376 PMC4754196

[109] De Meersman RE. Heart rate variability and aerobic fitness. Am Heart J (1993) 125:726–31. 10.1016/0002-8703(93)90164-5 8438702

[110] Aubert AE Seps B Beckers F Heart rate variability in athletes. Sports Med (2003) 33:889–919. 10.2165/00007256-200333120-00003 12974657

[111] Bigger JT Fleiss JL Steinman RC Rolnitzky LM Schneider WJ Stein PK. RR variability in healthy, middle-aged persons compared with patients with chronic coronary heart disease or recent acute myocardial infarction. Circulation (1995) 91:1936–43. 10.1161/01.CIR.91.7.1936 7895350

[112] Agelink M Boz C Ullrich H Andrich J. Relationship between major depression and heart rate variability. Clinical consequences and implications for antidepressive treatment. Psychiatry Res (2002) 113:139–49. 10.1016/S0165-1781(02)00225-1 12467953

[113] Liao D Cai J Brancati FL Folsom A Barnes RW Tyroler HA Association of vagal tone with serum insulin, glucose, and diabetes mellitus—The ARIC Study. Diabetes Res Clin Pract (1995) 30:211–21. 10.1016/0168-8227(95)01190-0 8861461

[114] Appelhans BM Luecken LJ. Heart rate variability and pain: associations of two interrelated homeostatic processes. Biol Psychol (2008) 77:174–82. 10.1016/j.biopsycho.2007.10.004 18023960

[115] Haensel A Mills PJ Nelesen RA Ziegler MG Dimsdale JE. The relationship between heart rate variability and inflammatory markers in cardiovascular diseases. Psychoneuroendocrinology (2008) 33:1305–12. 10.1016/j.psyneuen.2008.08.007 18819754 PMC4266571

[116] Tobaldini E Nobili L Strada S Casali KR Braghiroli A Montano N. Heart rate variability in normal and pathological sleep. Front Physiol (2013) 4:294. 10.3389/fphys.2013.00294 24137133 PMC3797399

[117] Conder RL Conder AA. Heart rate variability interventions for concussion and rehabilitation. Front Psychol (2014) 5:890. 10.3389/fpsyg.2014.00890 25165461 PMC4131496

[118] Crosswell AD Lockwood KG Ganz PA Bower JE. Low heart rate variability and cancer-related fatigue in breast cancer survivors. Psychoneuroendocrinology (2014) 45:58–66. 10.1016/j.psyneuen.2014.03.011 24845177 PMC4344376

[119] Shaffer F Venner J Heart rate variability anatomy and physiology. Biofeedback (2013) 41:13–25. 10.5298/1081-5937-41.1.05

[120] Beauchaine TP Thayer JF Heart rate variability as a transdiagnostic biomarker of psychopathology. Int J Psychophysiol (2015) 98(2 Pt 2):338–50. 10.1016/j.ijpsycho.2015.08.004 26272488

[121] Moss D Shaffer F The application of heart rate variability biofeedback to medical and mental health disorders. Biofeedback (2017) 45:2–8. 10.5298/1081-5937-45.1.03

[122] Dekker JM Schouten EG Klootwijk P Pool J Swenne CA Kromhout D. Heart rate variability from short electrocardiographic recordings predicts mortality from all causes in middle-aged and elderly men. The Zutphen Study. Am J Epidemiol (1997) 145:899–908. 10.1093/oxfordjournals.aje.a009049 9149661

[123] Fagundes CP Murray DM Hwang BS Gouin JP Thayer JF Sollers JJ III Sympathetic and parasympathetic activity in cancer-related fatigue: more evidence for a physiological substrate in cancer survivors. Psychoneuroendocrinology (2011) 36:1137–47. 10.1016/j.psyneuen.2011.02.005 21388744 PMC3128662

[124] Zhou X Ma Z Zhang L Zhou S Wang J Wang B Heart rate variability in the prediction of survival in patients with cancer: a systematic review and meta-analysis. J Psychosom Res (2016) 89:20–5. 10.1016/j.jpsychores.2016.08.004 27663106

[125] Aeschbacher S Schoen T Dörig L Kreuzmann R Neuhauser C Schmidt-Trucksäss A Heart rate, heart rate variability and inflammatory biomarkers among young and healthy adults. Ann Med (2017) 49:32–41. 10.1080/07853890.2016.1226512 27534940

[126] Nussinovitch U Elishkevitz KP Nussinovitch M Segev S Volovitz B Nussinovitch N. Reliability of ultra-short ECG indices for heart rate variability. Ann Noninvasive Electrocardiol (2011) 16:117–22. 10.1111/j.1542-474X.2011.00417.x 21496161 PMC6932379

[127] Munoz ML van Roon A Riese H Oostenbroek E Westrik I de Geus EJ Validity of (ultra-) short heart rate variability recordings. J Epidemiol (1997) 145:696–706. 10.1371/journal.pone.0138921 PMC4586373 26414314

[128] Shaffer F Shearman S Meehan ZM The promise of ultra-short-term (UST) heart rate variability measurements. Biofeedback (2016) 44:229–33. 10.5298/1081-5937-44.3.09

[129] Munoz ML van Roon A Riese H Thio C Oostenbroek E Westrik I Validity of (ultra-)short recordings for heart rate variability measurements. PLoS One (2015) 10:e0138921. 10.1371/journal.pone.0138921 26414314 PMC4586373

[130] McNames J Aboy M. Reliability and accuracy of heart rate variability metrics versus ECG segment duration. Med Biol Eng Comput (2006) 44:747–56. 10.1007/s11517-006-0097-2 16960742

[131] Bland JM Altman DG. Measuring agreement in method comparison studies. Stat Methods Med Res (1999) 8:135–60. 10.1177/096228029900800204 10501650

[132] Myles PS Cui J Using the Bland–Altman method to measure agreement with repeated measures. Br J Anaesth (2007) 99:309–11. 10.1093/bja/aem214 17702826

[133] Berkoff DJ Cairns CB Sanchez LD Moorman CT. Heart rate variability in elite American track-and-field athletes. J Strength Cond Res (2007) 21:227–31. 10.1519/R-20135.1 17313294

[134] Seppälä S Laitinen T Tarvainen MP Tompuri T Veijalainen A Savonen K Normal values for heart rate variability parameters in children 6-8 years of age: the PANIC Study. Clin Physiol Funct Imaging (2014) 34:290–6. 10.1111/cpf.12096 24666688

[135] Kuo TB Lin T Yang CC Li CL Chen CF Chou P. Effect of aging on gender differences in neural control of heart rate. Am J Physiol (1999) 277(6 Pt 2):H2233–9. 10600841 10.1152/ajpheart.1999.277.6.H2233

[136] Rennie KL Hemingway H Kumari M Brunner E Malik M Marmot M. Effects of moderate and vigorous physical activity on heart rate variability in a British study of civil servants. Am J Epidemiol (2003) 158:135–43. 10.1093/aje/kwg120 12851226

[137] Britton A Shipley M Malik M Hnatkova K Hemingway H Marmot M. Changes in heart rate and heart rate variability over time in middle-aged men and women in the general population (from the Whitehall II Cohort Study). Am J Cardiol (2007) 100:524–7. 10.1016/j.amjcard.2007.03.056 17659940 PMC11536487

[138] Drury RL Ginsberg JP Unpacking health as a complex adaptive system: new and emerging technologies for integrative ambulatory autonomic self-regulation is a catalyst in the synergy of data mining, cloud computing, machine learning and biosensors. Front Res Topic (2017) (in press).

[139] Bonnemeier H Richardt G Potratz J Wiegand UK Brandes A Kluge N Circadian profile of cardiac autonomic nervous modulation in healthy subjects: differing effects of aging and gender on heart rate variability. J Cardiovasc Electrophysiol (2003) 14:791–9. 10.1046/j.1540-8167.2003.03078.x 12890036

[140] Aeschbacher S Schoen T Dörig L Kreuzmann R Neuhauser C Schmidt-Trucksäss A Heart rate, heart rate variability and inflammatory biomarkers among young and healthy adults. Ann Med (2017) 49:32–41. 10.1080/07853890.2016.1226512 27534940

[141] Lehrer PM Gevirtz R Heart rate variability: how and why does it work? Front Psychol (2014) 5:756 10.3389/fpsyg.2014.00756 25101026 PMC4104929

[142] Moss D Shaffer F Foundations of Heart Rate Variability Biofeedback: A Book of Readings. Wheat Ridge, CO: Association for Applied Psychophysiology and Biofeedback (2016).

[143] Tan G Shaffer F Lyle R Teo I Evidence-Based Practice in Biofeedback and Neurofeedback. 3rd ed Wheat Ridge, CO: Association for Applied Psychophysiology and Biofeedback (2016).

[144] Zerr C Kane A Vodopest T Allen J Fluty E Gregory J HRV biofeedback training raises temperature and lowers skin conductance [Abstract]. Appl Psychophysiol Biofeedback (2014) 39:299 10.1007/s10484-014-92549

